# Microextraction Techniques with Deep Eutectic Solvents

**DOI:** 10.3390/molecules25246026

**Published:** 2020-12-19

**Authors:** Orfeas-Evangelos Plastiras, Eirini Andreasidou, Victoria Samanidou

**Affiliations:** Laboratory of Analytical Chemistry, Department of Chemistry, Aristotle University of Thessaloniki, 541 24 Thessaloniki, Greece; plasorfe@chem.auth.gr (O.-E.P.); eiriniia@chem.auth.gr (E.A.)

**Keywords:** deep eutectic solvents, microextraction, solid phase extraction, solid phase microextraction, stir bar sorptive extraction, liquid phase microextraction, liquid–liquid microextraction, single drop microextraction, dispersive liquid–liquid microextraction

## Abstract

In this review, the ever-increasing use of deep eutectic solvents (DES) in microextraction techniques will be discussed, focusing on the reasons needed to replace conventional extraction techniques with greener approaches that follow the principles of green analytical chemistry. The properties of DES will be discussed, pinpointing their exceptional performance and analytical parameters, justifying their current extensive scientific interest. Finally, a variety of applications for commonly used microextraction techniques will be reported.

## 1. Introduction

Sample preparation is considered to be the bottleneck of the whole analytical process, because it covers a plethora of operations that are essential to modify the sample, to make it amenable for chromatographic analysis, or to improve the analytical parameters, such as precision and accuracy. [1,2] Furthermore, it eliminates typical problems like possible interferences and low sensitivity. Samples can be considered as being made from two distinct parts, the analytes of interest and the matrix. The analytes of interest are the compounds to be determined, while the matrix is the rest of the sample, which not only does not necessitate analysis, but also may contain interfering substances. Sample preparation may focus on the analytes’ extraction, on the matrix transformation, or on both, in order to achieve dissolution, cleanup, preconcentration, or chemical modifications of the sample, so as to obtain better analytical results [3].

In recent years, more and more attention has been devoted to replacing conventional extraction techniques with the so-called “green” extraction techniques. This started with the introduction of green analytical chemistry (GAC) and the 12 principles, formulated by P. Anastas in 1998, the main concern of which was to create environmentally friendly analytical techniques, especially extraction techniques [4]. Greener approaches to extraction techniques include more inexpensive, quicker, and environmentally safe practices in environmental, clinical, and food analysis [5]. The adverse environmental impact of analytical procedures has been reduced in three different ways: reduction of the volume of solvents required in sample preparation; reduction in the amount and the toxicity of solvents and reagents employed in the measurement step, especially with automation and miniaturization; and the development of different direct analytical techniques not requiring solvents or reagents [6]. Thus, newly developed extraction techniques, which have a greener approach, are on the rise. Moreover, microextraction techniques, for example solid phase microextraction (SPME) and liquid phase microextraction (LPME), which will be discussed below, are considered to be green extraction techniques, owing to the use of very small quantities of solvents of the extraction phase in relation to the volume of the sample or to the solvent volume used in classical approaches [7].

The development and application of sustainable solvents has been a hot topic in different scientific and technological areas [8,9,10,11,12,13,14,15]. In this regard, remarkable advances towards the replacement of volatile organic solvents have been achieved by a group of organic salts with melting points below 100 °C, generally referred to as ionic liquids (ILs) [16]. Nevertheless, the problems with their biodegradability, toxicity, stability, and the expensive synthesis make them less than perfect solvents [17]. Therefore, deep eutectic solvents (DESs), were introduced in 2001 as an alternative to ILs. These showed a stronger ecofriendly profile, with easier and cheaper production, while having similar properties [18]. DESs contain large, asymmetrical ions that have low lattice energy and, thus, low melting points. They are often acquired by the complexation of a quaternary ammonium salt with a metal salt or hydrogen bond donor (HBD). The charge delocalization occurring through hydrogen bonding between, for instance a halide ion and the hydrogen-donor moiety, is responsible for the decrease in the melting point of the mixture, in relation to the melting points of the individual components [19,20]. Since 2001, many scientists around the globe pursed the utilization of DESs and published a variety of studies [21,22,23,24,25,26,27,28,29,30,31].

Our aim in this review will be towards the use of DESs in analytical extraction and microextraction techniques, while briefly presenting some frequently used DESs, their synthesis methods and their properties.

## 2. Synthesis and Properties of Deep Eutectic Solvents

### 2.1. Synthesis of Deep Eutectic Solvents

**Figure 1:**
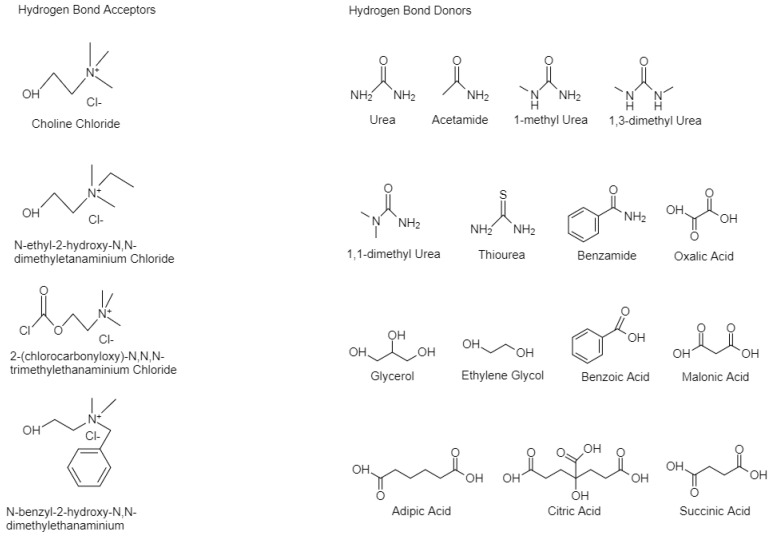
Structures of some halide salts (HBAs) and hydrogen bond donors (HBDs) used in the formation of deep eutectic solvents.

One of the approaches used to synthesize DESs is by mixing and heating two or more salts to form a homogenous solution. Typically, ambient temperature molten salts have been formed by mixing quaternary ammonium salts, a hydrogen bond acceptor (HBA) with metal salts [32,33]. Hence, four main different types of DES occur: I. quaternary salt with a metal chloride, II. quaternary salt with a hydrated metal chloride, III. quaternary salt (HBA) with an HBD compound and IV. metal chloride with an HBD. The respective general formulas of the four types are shown at Table 1. The first two types are used to synthesize hydrophilic DESs, whilst the other two for hydrophobic DESs [20,34]. Due to the stability in the aquatic solutions which type III and IV provide, they are often utilized in many papers, with choline chloride (ChCl) being the most used HBA. In Figure 1, some common HBA and HBD are shown.

### 2.2. Properties of Deep Eutectic Solvents

Various interactions (such as anion exchange, weak non-covalent interactions, π-π and/or hydrogen bonding) take place, amongst an HBD and an HBA in various combinations and molar ratios that contribute to some of the physicochemical properties discussed below [35]. Those properties are viscosity, density, conductivity, acidity, surface tension, volatility, and melting or freezing point. There is also an intensive study explaining the effect of hydrogen bonding proton transfer mechanism on the physical and chemical properties [36]. Other properties taken into account before selecting the optimal DES are biodegradability, toxicity, and thermal stability [37]. Moreover, both ILs and DESs can exhibit different physicochemical properties as binary mixtures, depending on the selected co-solvent, such as water or organic solvents. The interactions that may occur from the solute–solvent combination are defined as solvatochromic properties, which can further aid in the comprehension of chemical reactions through preferential solvation (PS) [38]. A useful tool to investigate the PS parameters and interactions, like hydrogen bond between molecules and ions, is the molecular dynamics (MD) simulation, which can be used as completion and confirmation of experimental results. For instance, Aryafard et. al. conducted an extensive investigation for three different DESs and their binary mixtures with polyethylene glycol (PEG 400) as co-solvent, through MD simulations analysis, while showing that PEG 400 can make a strong hydrogen bond with DESs [39].

## 3. Microextraction Techniques Using DES

### 3.1. Solid Phase Microextraction

#### 3.1.1. Solid Phase Extraction

Solid phase extraction (SPE) is an efficient technique commonly used to handle aqueous samples and extract semi-volatile or non-volatile analytes, due to it being easy, inexpensive, and possible to be automated. SPE was firstly introduced in the 1950s [40], and it uses appropriate sorbents so as to isolate the target compounds from the sample. Currently, there is a wide variety of available sorbents to cover almost all the possible interactions with the compounds. Nevertheless, analytes and interferents could coelute from the SPE sorbent because of the limited selectivity of conventional solid sorbents, such as silica-based, carbon-based, and clay-based resins [41,42]. Furthermore, due to the consumption of significant quantities of sample, organic solvents, and sorbents, the SPE technique is still improving [41].

There are many modifications to the conventional SPE technique, including magnetic SPE (MSPE), pipette-tip SPE (PT-SPE) and dispersive SPE (dSPE or DSPE) [43]. In MSPE, magnetic nanoparticles are utilized, such as magnetite (Fe_3_O_4_) and maghemite (γ-Fe_2_O_3_), and afterwards, they are embedded in a suitable coating of inorganic substances, mainly in graphene [44]. PT-SPE is a miniaturized form of SPE that resembles the procedure of conventional SPE in terms of conditioning, sample loading, washing, and elution [45]. DSPE is based on the dispersion of a solid sorbent in liquid samples for the extraction, isolation, and cleaning of various target compounds from complex matrices [46].

Jeong et al. presented the idea of tailoring and recycling of deep eutectic solvents as a sustainable and efficient extraction media to maximize the extraction efficiencies of several compounds found in ginseng. While experimenting with different solvents and ratios, they found that the combination of three effective DES components of sucrose, L-proline, and glycerol at a molar ratio of 1:4:9 provided the best results. The recovery of this study was determined to be 102.6 ± 4.1% (*n* = 5), and the recycled solvents’ extraction efficiencies were 91.9 (±2.9%), 85.4 (±2.3%), and 82.6 (±4.7%) for every recurring recycle, indicating that the DES can be recycled and reused at least three times, so as to achieve a high level of extraction yields [47].

N. Fu et al. experimented with environmentally friendly and non-polluting solvents, in order to extract polyphenols (such as protocatechuic acid, catechins, caffeic acid and epicatechin) from palm samples by using different ChCl DES. They tried eight different compounds with the ChCl to optimize the conditions of enrichment and purification of SPE, so as to acquire high yields. The optimal DES for the enrichment step was ChCl-FA, with a molar ratio of 1:1, and for the purification step, they utilized a ChCl-Ph DES-modified absorbent for the SPE cartridges and ChCl-Urea-H_2_O (1:1:5), ChCl-Gly (1:1), ChCl-FA-H_2_O (1:1:5), and H_2_O as eluents. The recoveries of the four analytes were 100% on the first cycle of usage of the DES, and they maintained a percentage between 60–117% throughout the five cycles studied in this work [48].

G. Li et al. tested DES modified molecular imprinted polymers (DES-MIPs) and DES modified non-imprinted polymers (DES-NIPs) for the purification of chlorogenic acid (CA) from honeysuckle. The preparation of these absorbents is explained in their paper. The DES used in this work had a ChCl base and glycerol, with a molar ratio of 1:2. The recoveries of MIPs, NIPs, DES-MIPs, and DES-NIPs were determined by the comparison of the peak areas of CA before and after the usage of SPE and were found to be 69.34%, 60.08%, 72.56%, and 64.79%, respectively [49].

Wang et al. utilized graphene (G) and graphene oxide (GO) whilst modifying them with DES and supporting them on silica as absorbents for SPE, for the preconcetration and extraction of three chlorophenols (4-chlorophenol, 2,4-dichlorophenol, and 2,4,6-trichlorophenol) from environmental water samples. The DESs used in this work were ChCl with FA, acetic acid, propionic acid, urea, Gly, ethylene glycol, and 1,4-butanodiol at the molar ratios of 1:2 [50].

In another study, G. Li et al. worked with Fe_3_O_4_/MIPs which were modified by ChCl based DES for the swift purification of alkaloid isomers (theophylline and theobromine) from green tea samples. MSPE was used as the purification method of the two compounds. The washing solution used was a mixture of methanol and water (80:20 *v/v*), and the eluent consisted of methanol/acetic acid (80:20 *v/v*) at pH 3 and with a volume of 4 mL. The recoveries of theophylline and theobromine in green tea were 87.51% and 92.27%, respectively. The optimal DES for this study was ChCl-Urea (1:2) [51].

X. Li et al. applied ternary DES in MIPs for the purification of levofloxacin, a known quinolone. The mixture of DES contained Caffeic Acid, ChCl, and FA at different molar ratios and were prepared by the heating method. The DES that exhibited the highest recovery rate (91.4%) was the one with a molar ratio of 1:3:1.5 [52].

Chen et al. utilized ternary hydrosulphonyl-based DES (THS-DES) modified by magnetic GO for the removal of mercury (Hg^2+^) from water, by using MSPE. The THS-DES was consisted of ChCl-itaconic acid-3-mercaptopropionic acid (2:1:1), and the removal efficiency achieved was 99.91% under optimized conditions. The THS-DES@M-GO absorbent retained a high removal efficiency of 90.23% even after seven MSPE cycles [53].

Ma et al. synthesized two types of MIPs based on magnetic chitosan with different DES (Fe_3_O_4_-CTS@DES-MIPs) and applied them as adsorbents in MSPE for the selective separation of catechins ((+)-catechin, (−)-epicatechin, and (−)-epigallocatechin gallate) in black tea samples. The two DESs were formed by ChCl/methacrylic acid (1:2 molar ratio) and betaine/methacrylic acid/H_2_O (1:2:1). The DES which gave the highest recovery rates was the first one, showing outstanding recognition, selectivity, specificity and magnetism. The recovery rates of the three catechins were 95.4%, 92.4%, and 90.3%, respectively [54].

Another study by Ma et al. that utilized MSPE was about extracting catechins from green tea, by using DES-MIP as a functional monomer and magnetic molybdenum disulfide as a base (Fe_3_O_4_@MoS_2_@DES-MIP). The catechins examined were the three mentioned above plus (−)-epigallocatechin and (−)-epicatechin gallate. The DES consisted of vinyl pyrrolidone and malonic acid (molar ratio 1:1). Both intra-day and inter-day yielded recoveries of the five analytes between 80–98% [55].

Liu et al. used DES modified graphene for the determination of sulfamerazine in river water samples, with the PT-SPE as the extraction technique. In particular, they tested the use of ChCl as a base and ethylene glycol, Gly, or urea as an HBD (at a 1:2 molar ratio with every solvent combination). Afterwards, they synthesized the DES-graphene and placed it in a 100 μL pipette tip using degrease cotton at both ends as frits. A 1 mL PT was inserted into the 100 μL PT, and the adsorbent was activated with 1 mL methanol and 1 mL of water. The extraction recovery by DES-G was 95.38%, whilst the G presented a 26.98% recovery rate [56].

Sun et al. utilized DES modified by a heteroatom doped GO for the application of PT-SPE and the extraction of two flavonoids, myricetin and rutin. Two different types of GO were prepared, one doped with nitrogen (N-GO), and the other with boron (B-GO). Subsequently, they prepared the N-GO-DES and B-GO-DES, with the DES comprising of ChCl-ethylene glycol. Finally, N-GO-DES was selected as the optimum adsorbent for this method, due to its permeability and high recoveries (that of myricetin was in the range of 70.61–99.77%, and rutin in between 76.58–98.14%) [57].

Yousefi et al. utilized dSPE for the ultra-trace analysis of organochlorine pesticides by using hydrophilic DES magnetic bucky gels (MBG) and magnetic multi-walled carbon nanotube nanocomposites (MMWCNT), prior to GC-μECD. The DES acted as a carrier and, at same time, as a disperser of the MMWCNTs and MBG. Thus, they opted for ChCl:Urea, 1:2 molar ratio, which had proper stearical interactions with MMWCNT, and it was miscible with water, so as to improve the dispersion of the sorbent. The LOD of this method was found to be 0.04–0.27 ng/L, and the enhancement factors were between 270–340, which are better than other techniques cited in the study [58].

Lamei et al. also used dSPE with magnetic GO nanoparticles coated with DES and ultrasound for the preconcentration of methadone in water and biological samples followed by GC-MS and GC-FID. The developed magnetic GO-DES (Fe_3_O_4_@GO-DES) was dispersed by ultrasound in the mixture of deionized water, NaOH and the sample solution that contained methadone to extract and preconcentrate the analyte. The DES studied in this work were ChCl with TNO (5,5,8,8-tetramethyl-5,6,7,8-tetrahydronaphthalen-2-ol), glycerol, and phenol at different molar ratios. The most efficient of the three was TNO, at molar ratio of 1:2 (ChCl:TNO), due to the high recoveries it provided. The LOD by using this method with GC-FID was 0.8 μg/L, whilst with GC-MS it was 0.03 μg/L. The recoveries of methadone in different samples were as follow: in distilled water, 98.4–101.5%; in urine, 95.3–99.3%; and in plasma, 95.1–96.4% [59].

Zarei et al. applied DES based magnetic colloidal gel for dSPE of ultra-trace amounts of some nitroaromatic compounds in water samples, followed by GC-μECD. The magnetic colloidal gel consisted of MMWCNTs and a deep eutectic solvent (ChCl:urea, ChCl:phenol, ChCl:acetic acid, or ChCl:gly at a molar ratio of 1:2). The optimal DES that provided the highest extraction efficiency was ChCl:urea. Then, the mixture was sonicated for half an hour to obtain the black paste or gel. The compounds examined in this work were 2-NT (2-nitrotoluene), 3-NT (3-nitrotoluene), 2,6 DNT (2,6-dinitrotoluene), 2,4 DNT (2,4-dinitrotoluene), and TNT (2,4,6-trinitrotoluene). The LODs of each compound analyzed with this method were 12.4 ng/L, 8.9 ng/L, 0.9 ng/L, 1.6 ng/L, and 0.8 ng/L, respectively, and the relative recovery rates were 90–105%, 90–110%, 95–110%, 90–110%, and 95–107%, respectively, for each compound in the different water samples [60].

Majidi et al. developed an alcohol-based DES as a carrier of SiO_2_@Fe_3_O_4_, a sorbent used in magnetic d-μSPE. The aim of this study was to apply the above for the preconcentration and determination of morin in grape and apple juices, in diluted and acidic extracts of dried onion and in green tea infusion samples. Different molar ratios of tetramethylammonium chloride and EG were tested, and the optimal ratio was 1:3. The addition of 20% *v/v* methanol in the DES substantially enhanced the extraction efficiency of morin. At optimal conditions, the LOD of the analyte was observed as 0.91 μg/L, while the recoveries of morin were 82.80–90.56% in apple juice samples, 91.06–95.56% in commercial red grape juice samples, 93.00–98.93% in onion samples, and 94.26–95.30% in green tea samples [61].

Li G. et al. used a mixture of three DES in MIPs for the separation of two xanthines (theobromine and theophylline), (+)-catechin hydrate and caffeic acid from green tea samples via d-μMSPE. The ternary DES synthesized were ChCl-OA-EG, with molar ratios of 1:1:1, 1:1:2, and 1:1:3, ChCl-OA-Gly (1:1:1) and ChCl-Caffeic Acid-EG (1:1:1). ChCl-OA-Gly (1:1:1) gave the highest percentages of recovery for the four analytes, being 89.72–92.25% for theophylline, 87.15–91.86% for theobromine, 87.16–90.17% for (+)-catechin hydrate, and 86.17–92.32% for caffeic acid [62].

Lastly, in an additional study, Li G. et al. synthesized hydrophilic MI chitosan (HMICS) based on DESs that were used as a template and as a functional monomer for the enrichment of gallic acid from red ginseng tea samples by SPE. From the three different molar ratios studied in this work, the best was ChCl-Gallic acid (1:2), as it was the only one that had a stable form. The adsorption capacity of gallic acid on HMICS with DES was 10.13 mg/g, 2.36 times higher than that of NICS (non-imprinted chitosan) without DES [63].

#### 3.1.2. Solid Phase Microextraction

Solid phase microextraction (SPME) is a sample preparation technique developed by Pawliszyn in 1990, where a fiber, with a small diameter, coated with a sorbent, is placed in aqueous solutions, so as to extract the analytes [64]. There are mainly two approaches: headspace SPME (HS-SPME), when the coated fiber was exposed to the gas phase, absorbing volatile compounds in the headspace of gaseous, gas or liquid samples, and direct immersion SPME (DI-SPME), in which the fiber is immersed in a small volume of the liquid sample, extracting non-volatile and semi-volatile analytes. When the equilibrium between the fiber and the analytes is completed, the SPME fiber is inserted into a GC for thermal desorption or into a desorption solvent to a LC [65]. Other approaches to this technique are thin-film SPME (TF-SPME), in-tube SPME, hollow fiber SPME (HF-SPME), and in-needle SPME [43].

Wang et al. utilized the in-tube method of SPME, as well as a poly-DES monolithic column coupled online to HPLC for the determination of anti-inflammatory and non-steroidal drugs (ketoprofen, flurbiprofen and diclofenac). In fact, they prepared a polymer monolithic column based on the DES monomer of ChCl and itaconic acid, (molar ratio 3:2) and connected it on the six-port valve of the HPLC, in order to build up an online SPME-HPLC device. The linearity of the proposed method was proved by a determination coefficient of 0.9997 in the range of 0.05–50 ng/mL for the first two compounds, and 0.25–500 ng/mL for sodium diclofenac. The LODs are 0.01 ng/mL, 0.01 ng/mL, and 0.05 ng/mL, respectively, and the recoveries of these drugs in lake water samples are 91.6–113.7% for ketoprofen, 87.3–109.5% for flurbiprofen, and 91.4–109.2% for sodium diclofenac, while, in human plasma samples, the recovery rates were 84.5–105.5%, 84.7–104.5%, and 87–99.9%, respectively, for each NSAID [66].

Li Tiemei et al. produced three hydrophobic DES, consisting of ethyl 4-hydroxybenzoate and methyl trioctyl ammonium chloride, with the molar ratios of 2:1, 1:1, and 1:2, which were used as an effective additive of the sol-gel sorbent coating of a PDMS fiber. This additive could create plenty of neat pores in the surface of the PDMS fiber, substantially improving its extraction efficiency. The new PDMS-DES fiber was evaluated by HS-SPME and GC-FID for the determination of VOCs, such as toluene, ethylbenzene, and o-xylene. The optimal molar ratio of the three that were tested was 2:1, as it exhibited the highest extraction efficiency. The linearity ranges of the new fiber were between 10–1000 μg/L, and the LOD was in the range of 0.005–0.025 μg/L [67].

Recently, Mirzajani et al. fabricated hollow fiber and monolithic fiber based on MOFs-DES/MIPs for the extraction of phthalate esters in yoghurt, bottled water, and soybean oil under termed hollow fibers liquid membrane-protected SPME (HFLMP-SPME), followed by GC-FID. Under optimal conditions, LODs of dimethylphthalate, diethylphthalate, di-isobutylphthalate, and n-dibutylphthalate were 0.008 μg/L, 0.012 μg/L, 0.030 μg/L, and 0.030 μg/L, respectively, while the mean recoveries of the analytes varied from 95.5–100.0%. The DES consisted of ChCl-Gly (2:1), which was then used for the preparation of the MOF-DES/MIPs SPME fibers [68].

#### 3.1.3. Stir Bar Sorptive Extraction

Stir bar sorptive extraction (SBSE) was originally developed and published by Baltussen et al. in 1999. They presented a novel approach on sample enrichment by using the sorbent PDMS coated onto stir bars with a length of 10 mm and 40 mm (coated with 55 μL and 219 μL of PDMS liquid phase, respectively). The former is more suitable for stirring sample volumes from 10–50 mL, and the latter is suited for volumes up to 250 mL [69]. When the adsorption process completes, the back-extraction of the analytes takes place, by liquid desorption, into an appropriate organic solvent. Benedé and her colleagues introduced a combination of SBSE and d-μSPE in 2014, which was called stir bar sorptive-dispersive microextraction (SBSDME). They utilized a neodymium-core stirring bar physically coated with a hydrophobic magnetic nanosorbent (MNP). Depending on the stirring speed, the magnetic nanosorbent either acts as a coating to the stir bar, rendering extraction feasible like SBSE, or as a dispersed medium for the extraction of the target analytes, like d-μSPE. To extract the analytes from the sample, the same methodology with SBSE was carried out [70].

In a study, Zarei et al. prepared a DES magnetic nanofluid, which was coupled with SBSDME as a hyphenated sample technique for the efficient extraction of ultra-trace nitroaromatic explosives in wastewater. Out of the four DESs, the one that was the most compatible for this work was ChCl:Resorcinol (1:2 molar ratio). The extraction solvent used was acetone, and the extract was then injected into the GC instrument for analysis of the compounds. The LOD of the analytes were 4.92 for 2-NT, 3.19 for 3-NT, 0.25 for 2,6-DNT, 0.45 for 2,4-DNT, and 0.22 ng/L for TNT. The related linear ranges were between 20–1500 ng/L, 15–1500 ng/L, 1–1500 ng/L, 2–1500 ng/L, and 1–1500 ng/L, respectively, for each target compound. It is also mentioned that the developed method is both user and environmentally friendly, according to the principles of the green analytical chemistry [71].

Nemati et al. developed a SBSE method coupled to solidification of floating droplets in DLLME based on DESs for the effective derivatization and simultaneous extraction of acidic pesticides from tomato samples. Initially, the target compounds are adsorbed on a coated stir bar from tomato juice filled in a narrow tube. Then, the stir bar is removed upon extraction and put in a water-miscible DES (ChCl:EG 1:2) to elute the analytes. Finally, a derivatization agent and a water-immiscible DES (ChCl:Butyric acid 1:2) are added to the eluent. The LODs of the five acidic pesticides were 7 ng/L for dalapon, 9 ng/L for MCPA, 14 ng/L for 2,4-dichlorophenoxyacetic acid 7 for fenoxaprop, and 11 ng/L for haloxyfob. Lastly, their mean relative recoveries were 92–95%, 94%, 93–95%, 92–98%, and 94–99% for each compound, respectively [72].

### 3.2. Liquid Phase Microextraction

Liquid phase microextraction (LPME) is a well-known term referring to a group of miniaturized techniques, based on the fundamentals of liquid–liquid extraction, while overcoming many of its drawbacks. LPME has been developed at the late 1990s, following the principal introduction to microextraction after the establishment of SPME [73,74]. Those techniques focus on the extraction and enrichment of analytes during the sample preparation with the use of fewer resources, primarily by reducing the quantities of solvents to less than 100 μL, thereby harmonizing with the principles of green analytical chemistry (GAC). LPME can be classified into three different types: (1) hollow fiber liquid phase microextraction (HF-LPME), (2) single drop microextraction (SDME), and (3) dispersive liquid–liquid microextraction (DLLME). In addition to the tendency of reducing the use of solvents and the produced waste, in recent years, there have been new applications for specific techniques that include green solvents, such as ionic liquids and deep eutectic solvents, in order to substitute toxic organic reagents and minimize the cost [75,76].

In the recent years, there are many reports for LPME combined with DESs for the extraction of several compounds, with different applications based on the three different categories mentioned previously. One of the first applications of DES with LPME was introduced by Karimi et al. for the extraction, preconcentration and determination of lead and cadmium in edible oils, coupled with electrothermal atomic absorption spectrometry (ETAAS). The selected DES was a mix of ChCl-Urea (4:1 molar ratio) mixed with a 2% solution of nitric acid. The limits of detection resulted to be 0.008 ng g^−1^ for Pb and 0.0002 ng g^−1^ for Cd, results that were comparable or even better than those of similar methods. Additionally, the method was applied to edible oils, such as olive oil, sunflower oil, sesame oil, and soybean oil, with good recoveries (95–104%) from spiked samples [77].

Furthermore, the utilization of DES on ultrasound assisted LPME is substantial, with most applications referring to the determination of metals in water and food samples. The ultrasonication effect is used so that the aggregated DES droplets will gradually break into tiny droplets, while the use of an emulsifier for further enhancement of the process (usually with addition of THF) is possible too and is also referred to as emulsification liquid phase microextraction (ELPME). The stage that follows is centrifugation to achieve separation between the aqueous and DES rich phase. The DES phase is isolated, filled with extra volume of a proper solvent, if necessary, and analyzed with the selected method. The main combination chosen as an extraction solvent is ChCl-phenol at molar ratios of 1:3 and 1:4, applied to different samples for the determination of several analytes such as: cobalt Co (II) in a pharmaceutical supplement and tea samples by a microsample injection system, coupled with flame atomic absorption spectrometer (MS-FAAS) [78], Cr(III), and Cr(VI) in water samples with MS-FAAS [79], malachite green (MG) in farmed and ornamental aquarium fish water samples by UV-VIS spectrometry [80], cadmium (Cd) in water and ice tea samples by electrothermal atomic absorption spectrometry (ETAAS) [81], Patent Blue V analysis in syrup and water samples by UV-VIS [82], Hg^2+^ and CH_3_Hg^+^ in water samples and freshwater fish samples with ETAAS [83], and pesticide residues in different traditional Chinese medicines (TCMs) with HPLC-DAD [84].

#### 3.2.1. Hollow Fiber Liquid Phase Microextraction

Hollow fiber LPME (HF-LPME) was firstly introduced by Pedersen-Bjergaard and Rasmussen as a liquid–liquid–liquid microextraction technique, prior to capillary electrophoresis for the preconcentration of methamphetamine in biological samples [85]. This procedure followed the principles of supported liquid membrane (SLM), however instead of a membrane, a polypropylene hollow fiber can be utilized and immobilize the organic phase into its porous. HF-LPME can be divided into two different modes, two- and three-phase. In the two-phase mode, the analytes are extracted from an aqueous sample stream (referred as donor solution) into an organic solvent, which should be water immiscible and immobilized in the wall pores and inside the lumen of the hollow fiber. In the three-phase mode, there is an acceptor phase (usually aqueous) inside the lumen of the hollow fiber, while the analytes are extracted through a thin layer of organic solvent in the hollow fiber porous, without direct contact with the sample. In both modes, either the extraction solvent (in the two-phase mode) or the acceptor phase located in the lumen of the HF, can be collected, and injected in the detection system. [86] HF-LPME normally uses hydrophobic organic solvents as extraction solvents, however, DES-based methods can be used as an additional novelty within the concept of GAC.

One of the first applications of DES was presented by Alavi et al. with the development of a Carrier-mediated hollow fiber liquid phase microextraction (CMHF-LPME). In this extraction procedure, the fiber lumen was filled with a DES consisted by ChCl–Urea at 1:2 molar ratio, together with KClO_4_ as the receiving phase, and an organic solvent was submersed to soak fiber’s porous. The extract was removed from the hollow fiber, diluted with ultrapure water, and injected into ETAAS. The method was applied for the determination of lead content in opium samples as adulteration and mainly in whole blood of addicts. The LOD and LOQ were determined and compared to other methods (0.1 ng/mL and 0.4 ng/mL, respectively), while there were references in the literature with better values, the method excels in terms of low cost, reduced toxicity, and simplicity. Moreover, the obtained relative recoveries, through the determination of Pb in spiked samples, were within the range of 95.8–105.6% [87].

A similar method was developed by Khataei et al., where DES was used as an acceptor phase in three-phase HF-LPME. The DES filled the hollow fiber’s lumen and consisted of methyltriphenylphosphonium iodide Me (Ph)_3_PI and EtGly at a 1:4 molar ratio, with the addition of 20% *w/v* MeOH and 2.5% (*w/v*) salt to enhance the extraction efficiency. The main goal was to extract and preconcentrate two synthetic steroidal hormones dydrogesterone (DYD) and cyproterone acetate (CPA) from urine and plasma samples prior to HPLC-UV. Although the method is based on a simple detection instrument (UV-VIS), LODs were low, in the range of 0.5–2 μg/L. The obtained relative recoveries of spiked samples were between 85.9 and 117.5%, which indicates the applicability of the method to biological samples that require complicated sample preparation [88].

Another modification of HF-LPME-DES based on SLM was described by Rajabi et al., where the lumen of hollow fiber was filled with acceptor phase and DES was used as an extraction solvent to fill the pores of HF membrane. The proposed mixture was synthesized by ChCl and 1-phenylethanol and the extraction procedure was based on the analytes’ penetration into the HF porous, followed by back extraction into the aqueous acceptor phase with fixed pH at 2.50. The aim was to analyze traces of antiarrhythmic drugs in pharmaceutical wastewater, human plasma, and urine by HPLC-UV with LOD values between 0.3 and 0.8 ng mL^−1^, because of the method’s ability to clean-up and preconcentrate the target analytes [89].

Among the different applications about three-phase HF-LPME, references of Xue et al. and Zhang et al. are included, which focus on quantification of different analytes in traditional Chinese medicine (TCM). Xue utilized a MTAC-glycerol DES (1:3 molar ratio) as an acceptor phase in a U-configuration three phase HF-LPME. DES was diluted in methanol (9:1, *v/v*) to enhance the extraction efficiency and filled the hollow fiber, which was initially immersed in a mixture of alcohols (n-heptanol/n-nonanol, 7:3, *v/v*) to form a SLM in the wall pores. Upon the completion of the extraction, the acceptor phase is transferred to an Eppendorf tube, mixed with methanol for washing the tube, vortexed, and injected into HPLC. The method was successfully applied to dry powder of four medicinal herbs for the determination of their active compounds such as hesperidin, honokiol, shikonin etc. The detection limits of the proposed method were 0.3–0.8 ng/mL, while the higher extraction efficiency and absence of salt were considered as advantages in comparison with 2p-HF-LPME [90]. On the other hand, in Zhang’s report, a tetrabutylammonium chloride (TBAC)-hexanoic acid (HA) DES with 1:3 molar ratio was utilized to impregnate the porous as extraction phase and 85% DES in methanol to fill the lumen of hollow fiber as an acceptor phase. Dilution in methanol was performed to overcome the problem of DES’s low viscosity. At the same time, during the optimization, addition of salt into the sample (20%, *w/v*) was considered necessary to achieve the maximum extraction efficiency. DES-HF-LPME was combined with HPLC-UV for the determination of four cinnamic acids in TCM, with simultaneous research of active compound-plasma protein binding rate of cinnamic acid derivatives, which proved to be a satisfactory separation. Additionally, the procedure provides low LODs (0.1–0.3 ng/mL) values and good repeatability [91].

#### 3.2.2. Single-Drop Microextraction (SDME)

The single-drop microextraction technique (SDME) was initially introduced in 1995 by Liu and Dasgupta, which involved extraction with the use of a single drop of water-immiscible organic solvent as a gas sampling, supported in the tip of a microsyringe needle, and immersed into a large flowing aqueous drop to extract ammonia and sulfur dioxide, followed by detection with a light-emitting diode (LED) photodiode detector [92]. This original SDME approach is also known as direct immersion (DI-SDME), and it can also be performed in the headspace of the sample (HS-SDME). In addition, a comparison of the introduced technique with SPME showed that they were comparable in terms of precision, sensitivity, and extraction time, although SPME was considered less complicated [93]. SDME can also be subdivided into categories according to the number of solvent phases, as in the case of HF-LPME: a two-phase mode and a three-phase mode (extraction from an aqueous phase to an organic phase, and further, into a second aqueous phase), which applicates in HS-SDME [94]. In case of SDME, one main factor that may affect the extraction efficiency is the type of solvent. Specifically, a high viscosity solvent will enable the suspension of larger and more stable drops at the tip of the needle, thus, DESs can be utilized due to their favorable characteristics, like high viscosity at room temperature, high thermal stability, and low volatility.

One of the first reports of coupling DES with SDME was presented by Tang et al., who used a DES of ChCl-ethylene glycol (EG) with molar ratio 1:4 for the extraction of three terpenoids from Chamaecyparis obtusa leaves by headspace-solvent microextraction (HS-SME) prior to GC-FID. In order to enhance the extraction procedure, the temperature was set at 100 °C, because the selected analytes (terpenoids) are volatile, and released into the headspace to a greater extent under high temperatures. The volume of extraction solvent was very small (2 μL), however the LODs were sufficiently good and the applicability to volatile compounds was proven [95]. A further related research for HS-SDME was contracted by Triaux et al., who used a DES of tetrabutylammonium bromide (N4444Br)-dodecanol (1:2) for the extraction of terpenes from six spices, such as cinnamon, cumin, thyme, prior to GC-MS. The extraction temperature was set at 80 °C, with 90 min extraction time, to allow for the absorption of target volatile analytes on the DES drop, fixed at 1.5 μL. The application to 27 different terpens showed that half of the studied compounds had LOQs lower than 2 μg/g, as in the case of menthone, caryophyllene, borneol, which agrees with Tang’s conclusions. Therefore, DES-HS-SDME is a simple, cheap, green, and efficient technique for the extraction of terpenes [96].

Other applications refer to DES with usage of different types of SDME, which continue to be rare until today, can be divided according to SDME’s categories and utilization of different technologies. For instance, Yousefi et al. combined a ChCl:2-Chlorophenol DES (1:2 molar ratio) as a base fluid and magnetic multiwall carbon nanotube nanocomposite (DES–MBG) to create a hydrophobic magnetic bucky gel, used as an extraction solvent. The nanofluid was characterized by high viscosity and magnetic susceptibility, which led to high stability of the micro-droplet that permitted extraction in high temperatures and fast agitation rates. A volume of 20 μL of DES-MBG was selected as the optimal acceptor phase volume, while after the extraction, the micro-droplet was dissolved in 50 μL of desorption solvent (acetonitrile). The developed procedure was applied to the determination of benzene, toluene, ethylbenzene, and xylene isomers as harmful VOCs in water and urine samples. Additionally, the limits of quantifications were low, comparable with other HS-SDME methods (from 0.17 to 3.20 ng/mL) [97]. In addition to with the previous reference, Zarei et al. considered the use of ferrofluid of montmorillonite nanocomposite and DES as an alternative solvent for the extraction and preconcentration of explosives in water with DI-SDME. DES was composed by DL-menthol-decanoic acid in 1:2 molar ratio and the ferrofluid was reported as MMT@Fe_3_O_4_-DES without the use of any additional stabilizer during the preparation. The DES-FF-DSDME procedure included 50 μL of the ferrofluid, which was isolated from the aqueous sample by rod magnet, salt addition of 10% (*w/v*) NaCl into the sample and acetonitrile as desorption solvent, where the micro-drop was dissolved. The proposed technique was coupled with HPLC-UV and the limit of detection was comparable with other existing methods, with the main advantage being the use of a low amount of an environmentally friendly extraction solvent [98].

Moreover, there are recent studies of different analytes extracted by HS-SDME. Abolghasemi et al. used a DES-HF-SDME for the extraction and preconcentration of triazole pesticides in orange juice and vegetables followed by GC-FID. The most effective extraction was obtained with 2 μL of ChCl:4-chlorophenol (1:2 molar ratio), with the addition of 10% NaCl (*w/v*) into the sample. The extraction temperature was set at 85 °C, to increase the distribution of analytes between the headspace and aqueous phase, with simultaneous optimization of pH, stirring speed, and extraction time. Lower LODs (ranging from 0.82 to 1.0 μg/L) and more extensive linear ranges were obtained and compared to methods that utilized more sensitive detection systems like MS [99]. Another study for semi-volatile analytes (polycyclic aromatic hydrocarbons (PAHs)) in water samples was introduced by Mehravar et al., where DES was used as an extraction media is HS-SDME prior to GC-MS. DES was composed by ChCl-Oxalic acid (1:2 molar ratio) with addition of 10% (*w/v*) NaCl. After the extraction, the microdroplet was transferred to a vial with 10 μL n-hexane and 10 μL water, to achieve compatibility with the GC system. Finally, the method produced satisfactory results in terms of linearity, LOD, LOQ, but also during the application on real samples, with almost quantitative recoveries for 16 different analytes (94.40–105.98%) [100].

#### 3.2.3. Dispersive Liquid–Liquid Microextraction (DLLME)

Dispersive liquid–liquid microextraction (DLLME), a subcategory of LPME, is a novel microextraction technique that was introduced in 2006 by Rezaee et al. [101] as an easy, simple, rapid, and cheap method for the extraction and preconcentration of organic compounds in water samples. This method is based on the use of a mixture, containing extraction and dispersive solvents, which is injected to an aqueous sample, followed by shaking. As a result, the extraction solvent disperses into the sample as fine droplets and forms a cloudy solution, where the analytes are transferred, successively recovered by centrifugation. The sediment, where the analytes remain, is collected with a micro syringe and can be analyzed by various analytical instruments [102]. DLLME is considered a green microextraction technique, due to the low volumes of solvents. However, chlorinated organic solvents might be considered a drawback, because they are classified as toxic for human health, harmful for the environment, and expensive. In that case, an innovative solution is the use of deep eutectic solvents as a greener extraction option for different samples and matrices [103].

The first use of DESs as an extraction solvent in DLLME was introduced by Farajzadeh et al., to extract and preconcentrate pesticides in aqueous samples, such as commercial fruit juices and vegetables, combined with GC-FID. An HDES from ChCl:4-chlorophenol, in 1:2 molar ratio, was produced. The LODs ranged from 0.39 to 3.1 ng/mL and RSDs were low, both considered remarkably acceptable in comparison with other methods, where mass spectrometry was used [104]. Afterwards, there were many reports, containing a variety of samples like food, environmental water, and biological fluids, but also proposing the combination of DESs with different types of DLLME, such as ultra-sound/ultrasonic assisted (UA-DLLME), air assisted (AA-DLLME), vortex assisted (VA-DLLME), and microwave assisted (MA-DLLME), which are also referred as liquid–liquid microextraction (LLME) techniques [105,106]. In every case of application, there is a variety of parameters, which can affect the extraction efficiency. For example, the type and volume of extracting and dispersive solvents, time of extraction, volume of sample, etc., may have significant role in the extraction result [107].

In case of UA-DLLME, where ultrasound is used to increase the efficiency and mass transfer rate between both phases, Wang et al. developed a method based on hydrophobic DES combined with HPLC-UV for the determination of benzophenones in swimming pool and river waters. The best extraction recovery was obtained with a mix of TAC with DecA at 1:3 molar ratio, with a recovery range set between 90.2 and 103.5%. The LODs varied from 0.15 to 0.30 ng/mL [108]. Altunay et al. used a NADES based UA-DLLME method to determine traces of Cu, Cd, and Pb in honey by FAAS. Methyl green (MeG^2+^) was used to form a complex (ion-pair formation) between metal ions and a ligand, to ensure their separation. NADES was obtained after mixing citric acid-sucrose (Cit-Suc) at a mole ratio of 3:2. Several conditions, such as the volume of the NADES, the type and volume of aprotic solvent to obtain a cloudy state, ultrasound time and temperature, sample volume, etc., had to be optimized before any application. The limits of detection and quantification were in the range of 0.23–0.87 μg/kg and 0.78–2.94 μg/kg for the analytes, respectively, and a range of 90.3–98.4% for the recovery [109].

Werner utilized non-toxic and water-immiscible DES with UA-DLLME with solidification of the aqueous phase (SAP), to preconcentrate aromatic amines in aqueous environmental samples prior to analysis by HPLC-UV. In this study, a new low-density DES was synthesized, consisting of tri-hexyl(tetradecyl)phosphonium chloride: decanol at a molar ratio of 1:2 ([P14,6,6,6] Cl: decanol, 1:2), with ultrasound used as a dispersing agent. In addition to the main optimized factors for DES use, the pH of the sample and salt addition were tested, and optimal results were found using 40 µL of DES, pH = 12 and 40 mg of NaCl. The method was used for analysis of 1-naphthylamine, 4-chloroaniline, and 2-chloroaniline in environmental samples. River water, sea water, lake water, and melted snow water were spiked with specific a volume of each analyte, with the determined recoveries in the range of 85.1–99.9% [110]. Werner also used a DES-based UA-DLLME, combined with SAP, prior to HPLC-UV for preconcentration and determination of heavy metals in river water, lake water, and well water. The proposed method used a new DES, prepared by mixing [P14,6,6,6] Cl and TSA at 1:2 molar ratio, with addition of salt (NaCl) in the microextraction step to expedite the separation. The limits of detection were 0.05 μg/L, 0.13 μg/L, 0.06 μg/L, and 0.11 μg/L for lead(II), cadmium(II), cobalt(II), and nickel(II), respectively. Additionally, the method was evaluated by testing spiked certified reference materials, where the recoveries were in the range 91.4–101.6% [111].

In addition to the application for environmental samples, there are references for food and biological samples, [112,113], such as Ji’s et al. research for determination of sulfonamides in fruit juices with HPLC-UV. In this experiment, 0.8 mL of a TAC and 2-octanol DES (1:2 molar ratio) was used as extraction solvent to preconcentrate sulfapyridine, sulfamethazine, and sulfamethoxine through UA-DES-LLME. DES was mixed with 5 mL sample, vortexed, and sonicated for 3 min to achieve emulsification. Samples were ready for injection into the chromatographic system after centrifugation and filtration of the upper layer. In this case, it has been proved that the simultaneous use of both ultrasound and vortex increases the extraction efficiency. After optimization, the method had the advantage of great linearity and low LOD (0.02–0.05 μg/mL), compared to conventional techniques like LLE, SPE, and QuEChERS. On the other hand, Liao et al. proposed a deep eutectic solvent-based method with UA-DLLME, to extract and determine four macrolide antibiotics, azithromycin, roxithromycin, clarithromycin, and tylosin tartrate in swine urine samples. A mixture of ChCl–Phenol (1:2 molar ratio) was employed as DES extractor, and THF was also used as a demulsifier solvent. The extraction efficiency was affected by pH and salt concentration, set to 9.0 and 20% (*w/v*), respectively. The extraction was followed by LC-MS/MS analysis, where LODs were in the range of 0.02–0.1 μg/L and relative recoveries from 80 to 93% for spiked samples, not less than those of other methods for macrolides, but its novelty focuses on the short time of sample preparation and chromatographic separation [113].

VA-DLLME is a microextraction method, where vortex agitation is used to extract analytes from large sample volumes (aqueous donor phase) to the smallest one of organic solvent (intermediate phase), which is a mild emulsification procedure, followed by back-extraction from the intermediate phase. This procedure follows up with the general advantages of DLLME, however, it does not require a dispersive solvent [114].

Zhang et al. developed a green DES-based VA-DLLME method for the analysis of nitrite in environmental water and biological samples prior to HPLC-DAD. The sample preparation requires a diazotization reaction of nitrite with p-nitroaniline and diphenylamine, followed by the addition of TAC and OA at molar ratio 1:2, which was used as an extraction solvent. After the optimization of certain factors, the LOD and LOQ were 0.2 and 1 μg/L. The method was applied to tap water, lake water, well water, human urine, and saliva samples spiked with specific quantities, with recoveries (%) ranging between 90.5 and 115.2 [115]. Faraji used DESs in a VA-DLLME for the simultaneous determination of two auxins (indole-3-acetic acid (IAA) and 1-naphthaleneacetic acid (NAA)) in tap water, apple juice, orange juice, and banana juice, followed by HPLC. A novel hydrophobic deep eutectic solvent was prepared by mixing trioctylmethylammonium chloride (TOMAC) and isoamyl alcohol with molar ratio of 1:4. During the microextraction procedure there was absence of salt and dispersing solvent. LOD was 0.2 μg/L and 0.3 μg/L for IAA and NAA, while LOQ was 0.6 μg/L and 0.9 μg/L in a water matrix. The reported LODs were significantly better in comparison with the data obtained by other methods. Additionally, the recoveries of spiked samples were excellent, in the range from 94.0 to 99.8% for both auxins [116].

Li et al. introduced a VA-LLME technique, where a low viscosity DES was used, in order to achieve direct injection to GC-MS. The selected DES was composed by n-hexyl alcohol and N4444Br (4:1 molar ratio) and 80 μL was mixed with 5 mL of spiked sample, shaken in vortex and centrifuged to isolate the DES-rich phase, prior to GC-MS. The method was used for the determination of residual phthalate esters (PEs) in the food-contacted plastic products, with limit of detections being as low as 1 µg L^−1^, succeeding good results with a simple, green, low-cost DES-DLLME method [117]. Additionally, Safavi et al. developed a VA-LLME procedure for the preconcentration and extraction of malondialdehyde (MDA) and formaldehyde (FA), after derivatization with 2,4-dinitrophenylhydrazine (DNPH), from three different samples (human urine, apple juice, and rainwater). A volume of 35 μL DES of decanoic acid and methyltrioctylammonium bromide (2:1 molar ratio) was mixed with derivatized samples, vortexed for 2.12 min at 3900 rpm and centrifuged twice to isolate the extraction phase. The isolated phase was diluted by ethanol to achieve compatibility with HPLC-UV. The detection limits of MDA and FA were found as 2.0 ng/mL and 10.0 ng/mL, respectively, while the clean-up effect during the application to real samples was satisfactory, as well as the relative recoveries [118].

Air assisted DLLME is an alternative green procedure, introduced by Farajzadeh et al., where air is used during the dispersion procedure, without addition of dispersion solvent, which eliminates some disadvantages like higher solubility, and allows better extraction performance. In particular, the fine droplets of HDES are formed through a continuous back and forth movement by using a syringe [119]. Ge et al. synthesized hydrophobic DESs based on DL-menthol, which were used for the first time in a microextraction technique (AA-DLLME) to determine six benzophenone-type (BP-type) UV filters in water samples (swimming pool water, river water, and wastewater samples) with HPLC-DAD. The best extraction was obtained using DL-menthol and decanoic acid at molar ratio of 1:1, with addition of 1% NaCl to enhance the extraction procedure. The method provided low LODs and LOQs (0.05–0.2 ng/mL and 0.2–0.5 ng/mL), and was tested with application on real samples, spiked at three different levels of concentration, where relative recoveries were in the ranges of 88.8–105.9% [120].

A further alteration in air assisted techniques was the development of an AA-LLME method coupled with GC-MS by Jouyban et al., where a ferrofluid (toner@Aliquat 336-DES ferrofluid) was prepared from toner powder and deep eutectic solvent composed by ChCl:stearic acid (1:2 molar ratio). Samples were mixed with 76 μL of ferrofluid, with the addition of 0.4 g of NaCl (8%, *w/v*) to increase the extraction efficiency. The procedure included six cycles of aspirating/dispersing to extract the analytes into the ferrofluid droplets, which were easily removed with a magnet and sonicated after dilution with 10 μL of n-hexane. The method provided wide linear ranges, low LODs and LOQs, and was successfully applied for the determination of 16 different PAHs in urine and saliva samples of tobacco smokers [121].

Recently, El-Deen et al. developed a green AA-DLLME method based on floating organic droplet solidification (SFOD) with ternary DES as an extracting solvent, consisting of fatty acids like nonanoic acid, decanoic acid, and dodecanoic acid (C9:C10:C12, at molar ratio 1:1:1) and 9.5% salt concentration (NaCl). In DLLME-SFOD, the selected extracting solvent floats on the surface of the aqueous phase, and then solidified at low temperature in an ice bath. The extraction method was successfully used for the enrichment of five endocrine disruptors (EDCs) from tap and river water, determined by HPLC-PDA. The detection limits were between 0.96 and 2.30 μg/L, comparable with other reports of DLLME in the literature, but in that case, the extraction time was greener, faster, with recoveries of up to 90% [122].

Microwave assisted DLLME was also reported, where a glass vial containing the sample and the extraction solvent is placed under microwave power. Torbati et al. synthesized water-miscible DES (ChCl:phenol at 1:2 mole ratio) for the extraction and preconcentration of seven selected herbicides from wheat samples by GC-MS. A main parameter that may attribute to the extraction efficiency is the microwave’s power, which was set at 180 W. Under the optimum conditions, the LODs and LOQs varied between 1.6–12 ng kg^−1^ and 5.5–42 ng kg^−1^ lower than those obtained with many other methods, while the recoveries were found to be 93–102% [123].

Overall, the use of DESs constitutes a major modification in DLLME, because they are considered a low cost and green option as extraction solvents. Continuous developments are often presented, including automation. Shishov et al. developed a fully automated DLLME system, with DES as dispersive solvent. The instrumentation includes a multiposition valve, a syringe pump combined with a magnetic stirrer and connected to a cell of UV-VIS detector for the determination of Cr (VI) in different beverages (still water, sparkling water, apple and cherry juices, and two carbonated drinks). The most effective composition was tetrabutylammonium bromide - formic acid of 1:4 molar ratio combined with 1-octanol as extraction solvent and 1,5-di-phenylcarbazide as a color-forming reagent. The LOD and LOQ were 0.2 μg/L and 0.6 μg/L, respectively, which can be compared to other methods that require longer analysis time, non-automated systems and higher costs [124]. Further progress for the application of DES in LPME methods is its in situ formation during the sample preparation procedure, as presented by Li et al. for the determination of four fluoroquinolones (FQs) in surface water by LC-UV. In particular, they used a centrifuge tube, where 10 mL of aqueous sample solution were mixed with the individual DES components (67.5 mg of thymol and 31.9 μL of heptanoic acid), which formed thymol-fatty acid HDES as a water-immiscible liquid droplet after shaking and incubation at 52 °C. After the in situ formation, the tube was automated vortexed for 1 min, centrifuged for 2 min and the final DES enriched phase was diluted in the mobile phase for injection. The method provided a limit of detection of 3.0 ng/mL, and allowed the determination of FQs through a simple and fast protocol, which requires about 8 min, without additional time consumption for the synthesis of DES [125].

Among the different applications of DESs in liquid phase microextraction, there are several research articles which include modifications based on its principles, referred as LLME alterations, which are summarized in Table 2. Their common feature is the mixing of liquid reagents for the pretreatment, with additional use of emulsification or dispersive solvent, and different sample preparation tools like shaker-assisted LLME with back extraction (SA-LLME-BE), homogeneous LLME (DES-H-LLME), vortex-assisted-emulsification DLLME (VA-E-DLLME), and temperature-controlled LLME (TC-LLME).

## 4. Conclusions

The use of DESs in analytical microextraction techniques is on the rise, due to the many benefits they provide, such as lower cost and easier synthesis than ILs and an environmentally friendly profile, because of the low toxicity reported, although they need further investigation. To this day, the number of HBAs and HBDs is quite limited, so more studies ought to be carried out to present a plethora of DESs available for use. Moreover, DESs are not commercially available yet, substantially affecting and further limiting their usage for routine analyses in industrial or certified laboratories.

The extraordinary high relative recoveries, selectivity, low LODs and decent repeatability they offer, render them appropriate for the determination and quantification of lots of compounds in either simple or complex matrices. As seen, most applications regard liquid phase microextractions rather than solid phase microextractions, because of their liquid nature, as it is simpler to use them as supporting solid adsorbents. The fact that the sample preparation of complicated matrices is of high interest makes them ideal for the research.

Hopefully, DESs will be available for purchase in the foreseeable future and will replace organic solvents in some analytical methods commonly used nowadays, while more studies are carried out about their properties.

## Figures and Tables

**Table 1:** The four types of deep eutectic solvents.

Type	Formula	Terms
I	Cat^+^X^−^ + zMCl_x_	M = Zn, Sn, Al, Ga, Fe, In
II	Cat^+^X^−^ + zMC_lx_ · yH_2_O	M = Co, Cu, Ni, Fe, Cr
III	Cat^+^X^−^ + zRZ	Z = OH, COOH, CONH_2_
IV	MCl_x_ + RZ = MCl_x−1_^+^ · RZ + MCl_x+1_^−^	M = Zn, Al and Z = OH, CONH_2_

Cat^+^ = any phosphonium, ammonium or sulfonium cation, X^−^ = a Lewis base, generally a halide anion, MCl_x_ = metal chloride, RZ = organic compound.

**Table 2:** Compilation of application of deep eutectic solvents (DES) in various types of liquid phase microextraction procedures.

Extraction Technique ^1^	Selected DES Solvent, Molar Ratio	Analytes/Sample Matrix	Additional Requirements	Analytical Technique	Ref.
SA-LLME	decanoic acid and methyl-trioctylammonium bromide, 2:1	Methylene Blue/Water	Mixture HCl solution (2 M) and ethanol (50:20 volume ratio) as stripping phase	UV-VIS	[126]
AA-LLME	borneol and decanoic acid, 1:3	Warfarin/Urine & Plasma	(−)	HPLC-UV	[127]
VA-EDLLME	ChCl and phenol, 1:2	Phthalates/Beverages	THF (as emulsifier agent)	HPLC-DAD	[128]
TC-LLME	phenyl salicylate (salol) and DL-menthol, 1:1	Pd/Environmental samples	Water bath at 65 °C for 1 min	ETAAS	[129]
UA-LLME	TBABr^2^ and 1-octanol, 1:2	Erythrosine/biological and pharmaceutical samples	(−)	UV-VIS	[130]
HLLME	thymol and hexanoic acid, 2:1	Levofloxacin & ciprofloxacin/Environmental water	KOH (6 M) to dissolve the DES & HCL (6 M) to break the homogenous state	HPLC-UV	[131]

^1^ Abbreviations of AA-LLME: Air Assisted Liquid Liquid Microextraction; HLLME: Homogenous Liquid Liquid Microextraction; UA-LLME: Ultrasound Assisted Liquid Liquid Microextraction; SA-LLME: Shaker Assisted Liquid Liquid Microextraction; TC-LLME: Temperature Controlled Liquid Liquid Microextraction; VA-EDLLME: Vortex Assisted Emulsification Dispersive Liquid Liquid Microextraction.

^2^ Tetrabuthylammoniumbromide.

## Abbreviations

AA-DLLME: Air Assisted Dispersive Liquid Liquid Microextraction
AA-LLME: Air Assisted Liquid Liquid Microextraction
CA: Chlorogenic Acid
ChCl: Choline Chloride
Cit-Suc: Citric acid-sucrose
DecA: Decanoic Acid
DES: Deep Eutectic Solvent
DI-SPME: Direct Immersion Solid Phase Microextraction
DI-SDME: Direct Immersion Single Drop Microextraction
DLLME: Dispersive Liquid Liquid Microextraction
d-μSPE: Dispersive Micro Solid Phase Extraction
dSPE: Dispersive Solid Phase Extraction
EG: Ethylene Glycol
ELPME: Emulsification Liquid Phase Microextraction
ETAAS: Electrothermal Atomic Absorption Spectrometry
FA: Formic Acid
FID: Flame Ionization Detector
FQ: Fluoroquinolones
G: Graphene
GAC: Green Analytical Chemistry
GC: Gas Chromatography
Gly: Glycerol
GO: Graphene Oxide
HBA: Hydrogen Bond Acceptor
HBD: Hydrogen Bond Donor
HDES: Hydrophobic Deep Eutectic Solvent
HF-LPME: Hollow Fiber Liquid Phase Microextraction
HF-SPME: Hollow Fiber Solid Phase Microextraction
HLLME: Homogenous Liquid Liquid Microextraction
HPLC: High Pressure Liquid Chromatography
HS-SDME: Headspace Single Drop Microextraction
HS-SPME: Headspace Solid Phase Microextraction
LC: Liquid Chromatography
LLME: Liquid Liquid Microextraction
LOD: Limit of Detection
LOQ: Limit of Quantification
LPME: Liquid Phase Microextraction
MA-DLLME: Microwave Assisted Dispersive Liquid Liquid Microextraction
MIP: Molecular Imprinted Polymers
MSPE: Magnetic Solid Phase Extraction
μSPE: Micro Solid Phase Extraction
MNP: Magnetic Nanosorbent
MOF: Metal Organic Frameworks
NADES: Natural Deep Eutectic Solvent
NSAID: Nonsteroidal Anti-Inflammatory Drug
OA: Oleic Acid
PAH: Polycyclic Aromatic Hydrocarbons
Ph: Phenol
PDMS: Poly(dimethylsiloxane)
PT-SPE: Pipette Tip Sold Phase Extraction
QuEChERS: Quick Easy Cheap Effective Rugged Safe Solid Phase Extraction
SA-LLME: Shaker Assisted Liquid Liquid Microextraction
SAP: Solidification of Aqueous Phase
SBSE: Stir Bar Sorptive Extraction
SDME: Single Drop Microextraction
SFOD: Solidification of Floating Organic Droplet
SLM: Supported Liquid Membrane
SPE: Solid Phase Extraction
SPME: Solid Phase Microextraction
TAC: trioctylmethylammonium chloride
TBAC: tetrabutylammonium chloride
TC-LLME: Temperature Controlled Liquid Liquid Microextraction
TCM: Traditional Chinese Medicine
THF: tetrahydrofuran
TF-SPME: Thin Film Solid Phase Microextraction
TNO: 5,5,8,8-tetramethyl-5,6,7,8-tetrahydronaphthalen-2-ol
TOMAC: trioctylmethylammonium chloride
TSA: thiosalicylic acid
UA-DLLME: Ultrasound Assisted Dispersive Liquid Liquid Microextraction
UA-LLME: Ultrasound Assisted Liquid Liquid Microextraction
VA-DLLME: Vortex Assisted Dispersive Liquid Liquid Microextraction
VA-EDLLME: Vortex Assisted Emulsification Dispersive Liquid Liquid Microextraction
VOC: Volatile Organic Compounds
VP: Vinyl Pyrrolidone
[P14,6,6,6]Cl: trihexyl(tetradecyl)phosphonium chloride
